# A comparative Analysis of *In Vitro* and *In Vivo* Efficacies of the Enantiomers of Thioridazine and Its Racemate

**DOI:** 10.1371/journal.pone.0057493

**Published:** 2013-03-07

**Authors:** Jørn B. Christensen, Oliver Hendricks, Shaswati Chaki, Sayanti Mukherjee, Ayan Das, Tapan K. Pal, Sujata G. Dastidar, Jette E. Kristiansen

**Affiliations:** 1 Department of Chemistry, University of Copenhagen, Copenhagen, Denmark; 2 King Christian X Hospital for Rheumatic Diseases, University of Southern Denmark, Gråsten, Denmark; 3 Department of Microbiology, Herbicure Healthcare Bio-Herbal Research Foundation, Kolkata, India; 4 Bioequivalence Study Center, Department of Pharmaceutical Technology, Jadavpur University, Kolkata, India; 5 Memphys Center for Biomembrane Physics, Department of Physics and Chemistry Odense, Denmark; Indian Institute of Science, India

## Abstract

A long list of chemotherapeutical drugs used in the treatment of the peripheral and the central nervous systems possess anti-microbial activity. Some of these neurotropic compounds are chiral, with the one stereo isomeric form exaggerating reduced neurotropism. This is the case for the levorotatory form of thioridazine. The phenothiazine thioridazine is an interesting compound, characterized by exhibiting a significant growth inhibiting activity on a wide array of micro-organisms. Thioridazine is characterized by another challenging feature, because the compound is concentrated in certain human tissue cells. The present study describes a comparative study of the two enantiomers as well as the racemic form of thioridazine. The study exploits the stereochemical aspect and the *in vitro* and *in vivo* potential of these compounds, with a focus on the effects on Gram negative organism *Salmonella enterica* serover Typhimurium. In summary, the results of this study yielded a significant antibacterial activity of all forms of thioridazine, indicating the levorotatory (–)- form to be superior in terms of both its *in vitro* and *in vivo* efficacies.

## Introduction

An antibiotic may be defined as a product from a microorganism capable of inhibiting the growth of another microorganism at distinctly low levels. The chemotherapeutics, on the other hand, are primarily synthetic compounds that are able to act on microorganisms in a very similar manner, but at much higher concentrations. It is now known that both antibiotics and antibacterial chemotherapeutics have lost the battle to a large extent in the fight against multidrug resistant (MDR) bacterial pathogens. However, intensive studies by various groups of scientists throughout the world have revealed that there are medicinal compounds used for the therapy of non-infectious pathology possess distinct antimicrobial properties [1]–[13]. These compounds are termed as non-antibiotics [10]. Non-antibiotics exhibit properties that render them important for the therapy of different MDR infections. Phenothazines being one of the most important group of non-antibiotics have been studied extensively for their antimicrobial potentiality [1]–[13], [14]. These non-antibiotics possess most of the characteristics of antibiotics and their antibacterial action can be further potentiated by suitable combinations [15]–[18].

The phenothiazine thioridazine (Tz) is a unique non-antibiotic which is highly bactericidal for Gram positive bacteria and acts as a bacteriostatic agent against Gram negative organisms [19].

Thioridazine is chiral and previous studies have reported that the levorotatory form (−) thioridazine is concentrated in human tissue cells at higher levels than the dextrorotatory form (+) [20]. Furthermore the (–) form of Tz has been reported to have less challenging pharmaco-dynamic activity, e.g. reduced blocking activity on centrally located dopamine D2-receptors than the (+)-form [21]. Several *in vitro* studies have shown another feature of racemic Tz.

The compound is concentrated in human macrophages and different tissue types, such as pulmonary epithelial cells.

Racemic Tz has a great potentiality for the therapy of MDR-tuberculosis since this compound is concentrated 100-fold in the human macrophages where the tubercle bacilli multiply and remain viable and where antibiotics fail to enter [22]. Furthermore racemic Tz has been shown to possess the capacity to lower the invasion ability of Gram positive and Gram negative bacteria in human epithelial cell lines [23]. Moreover racemic Tz proved to be highly efficient in disintegrating the invading cells of *Salmonella enterica* serover Typhimurium in mice at rather low levels [21]. The present study aims to define the specific antibacterial properties of the enantiomeric forms of thioridazine, e.g. the racemic, the (+)- and the (–)- compounds and clarify, whether there is a difference in the efficacy of the drug based on its stereoisomeric profile. In order to achieve this goal, we performed comparative *in vitro* and *in vivo* studies with two enantiomers along with the racemic compound available commercially (Sigma Chemicals, Denmark).

## Materials and Methods

### Bacteria

A total of 55 different bacteria belonging to both Gram positive and Gram negative types were taken for this study (Table 1).

**Table 1:** Minimum inhibitory concentration (MIC) of three optical forms of Tz with respect to different bacteria.

BACTERIA	MIC(µg/ml)		
	Racemic	(+)	(−)
*S. aureus* NCTC 6571, *V. cholerae* 569B, *V. cholerae* 1023	25	25	25
*S. aureus* NCTC 8530, *S. aureus* ATCC 25923, *S. dysenteriae* 7 NCTC 519, *Sh. boydii* NCTC 254, *V.cholerae* ATCC 14033,ATCC 14035, *V. cholerae* DN7	50	100	50
*Sh. flexneri* 4a NCTC 24, *Sh. sonnei* NCTC 9774, *V.cholerae* 713, 820	100	100	100
*S. aureus* ML 16, ML 152, ML 329, ML 358, *S. typhi* NCTC 59, *S. choleraesuis* NCTC 36, 37, *L. monocytogenes* NCTC 7973,NCTC 10351, NCTC 11994	200	200	100
*B. subtilis* ATCC 6633, *B. pumilus* NCTC 8241, *S. aureus* ML 266, ML 358, ML 422, *E. coli* K12 Row, *E. coli* C600, *S. berta* NCTC 69,*S. abony* NCTC 6017	200	200	100
*B. polymyxa* NCTC 4747, *B. licheniformis* NCTC 10341, *S. London* NCTC 76, *S. enterica* serovar Typhimurium NCTC 11, NCTC 74	500	500	500
*S. aureus* ML 277, *V. cholerae* 137/62	1000	1000	1000
*K. pneumoniae* ATCC 10031, *K. oxytoca* ATCC 130988	2000	2000	2000
*L. monocytogenes* AMRI 3, *A. boumannii* KPC 470, 517, *P. aeruginosa* ATCC 27853, ATCC 25619, C/1/5, Kr/8/89, BVC 1,2,3,4,5, APC1.	>2000	>2000	>2000

### Drugs

Racemic thioridazine (Sigma Chemicals, Denmark). The two enantiomers of thioridazine were prepared according to the procedure of Bourquin et al [22].

### Media

Liquid media were nutrient broth (NB; Oxoid), peptone water (PW) containing 1.0% peptone (Oxoid) and 0.5% NaCl and Mueller Hinton broth (MHB, Oxoid). Solid media were nutrient agar (NA, Oxoid), brain heart infusion agar (BHA), Oxoid and Mueller Hinton agar (MHA), Oxoid); pH was always maintained at 7.2 to 7.4.

### Inoculum

Each bacterium was grown in NA/MHA at 37°C, harvested at stationary phase and suspended in 5 ml sterile distilled water. Turbidity of each suspension was matched against 0.5 McFarland standard [14] along with a spectrophotometer at 625 nm corresponding to 2.4×10^5^ colony forming unit (CFU)/ml.

### Determination of Minimum Inhibitory Concentration (MIC) of the Enantiomers and the Racemate of Tz

This was carried out according to the guidelines of Clinical and Laboratory Standards Institute (CLSI) [24] by spotting 10^5^ CFU contained in a 2 mm loop from diluted 18 h broth cultures on plates containing 0 (control), 50, 100, 200,500, 1000 and 2000 µg/ml of a drug. The plates were incubated at 37^0^ C, observed for appearance of growth after 24 h and again after 72 h.

### Spectrophotometry

A stock solution containing 1 mg/ml of thioridazine HCl (Sigma), racemic mixture of thioridazine HCl, dextrorotatory thioridazine HCl (+) and levorotatory thioridazine HCl (−) were prepared by dissolving these separately in methanol. Each stock solution was further diluted to 10µg/ml of methanol. Aliquots of this solution were taken in a quartz cell and scanned for λ_max_ in the range of 200–600 nm using methanol as the blank in a double beam UV spectrophotometer (Jasco-V630). The maximum absorbance was determined by using Spectra manager (Version-2.05.03).

### HPLC Parameter

**Name of HPLC:** Jasco.

**Column:** C8, 250×4.6 mm, 5 µ particle size.

**Flow Rate:** 1.0 ml/min.

**Loop size:** 50 µ lit.

**UV Absorption:** 264 nm.

**Mobile Phase:** Methanol: Water containing 0.1% v/v phosphoric acid.

**Run Time:** 8 mins.

**Software used:** Clarity lite (Version: 2.6.4.402)

All the samples were prepared as per the above method and each sample was diluted by methanol (HPLC Grade).Water was used for the analysis was MilliQ water.

### Animal Experiments

Swiss albino male mice each weighing 18–20 gm were selected for this work. This study was approved by the Institutional Animal Ethics Committee (IAEC) of Jadavpur University and TAAB Biostudy Services. Animals were maintained under standard conditions of temperature (24±1°C) and relative humidity (50–60%) with a photoperiod of 14∶10 h of light:dark. Water and a dry pellet diet were provided ad libitum. The animals were checked regularly for their health and diet according to the rules and guidelines set by the Ethical Committee at definite intervals of time of 12 hr at 8 A.M. in the morning and 8 P.M. in the evening. The animals which showed symptoms of illness were carefully observed, identified and were separated from the healthy ones. These animals were not included in our experiments and were given proper treatment.

The intensity of virulence of infection caused by *Salmonella enterica* serovar Typhimurium 74 and the median lethal dose (MLD or LD_50_) of the mouse-passaged strain was as described earlier [14], [25]. Protective efficacies of the forms of Tz in mice infected with virulent *S. enterica* were carried out as described below. Four groups of animals with 60 mice in the control group and 20 mice each in the 6 experimental groups were taken. The control group received 0.1 ml of sterile saline while for each form of Tz there were 40 animals, in which 20 mice received 100 µg and the other 20 received 200 µg of the drug. After 3 h all the animals were infected with 50 MLD of virulent *S.enterica* 74 as described [25]. Protective capacity of all 3 forms of Tz was determined by recording the mortality of mice in the different groups upto 100 h after the challenge. The animals which survived after 100 hr of infection were euthanased with the help of cervical dislocation as suggested in the Ethical Committee. The end point for performing euthanasia was observation upto 100 hr with regular monitoring at 12 hr intervals. The animals which were euthanased prior to 100 hr were based on the condition of their health. Generally within 72 hr if they showed severe signs of illness, for example loss of appetite, weight loss, lack of movement, breathlessness, shivering etc. they were euthanased as advised by the veterinary doctor in the Ethical Committee The number of animals euthanased prior to 100 hr varied from 2–7 in each group. Assessment of the animals euthanized prior to 100 hr was monitored by the veterinary doctor after every 12 hr as mentioned 8 AM in the morning and 8 PM in the evening.

### Determination of the Effect of Treatment by 3 Forms of Tz on CFU of *S. enterica* 74 from the Liver, Spleen and Heart Blood of Infected Mice

In a separate experiment 4 groups of 5 mice each were given the following treatment: Group 1 was administered 0.1 ml saline while the other 3 groups received 200 µg each of racemic or (+) or (−) forms of Tz. After 3 h all the animals were challenged with 50 MLD of the same organism. After 18 h mice in each of the 4 groups were sacrificed by cervical dislocation (as recommended by the Ethical Committee) and their spleens and livers were aseptically removed; heart blood was drawn directly from the heart with the help of a micro-syringe for determination of CFU. The spleens and livers were homogenized separately in a tissue homogenizer maintained at 4°C and each specimen was processed for CFU counts.

### Statistical Analysis

The results were statistically evaluated by students ‘t’ test and χ^2^ test wherever applicable, Using freely available statistical software GraphPad (www.graphpad.com).

## Results

### Bacterial Inhibitory Spectra of 3 Different Forms of Tz

A total of 55 different Gram positive and Gram negative bacteria when tested against the 3 forms of Tz, racemic, (+) and (−), it was found that *S. aureus* NCTC 6571, *V. cholerae* 569B, 1023 could be inhibited at 25 µg/ml of each agent (Table 1). Among others it was found that strains of *S. aureus*, *V. cholerae* and shigellae were also sensitive to these agents MIC of racemic, (+) and (−) forms of Tz produced almost identical type of inhibition in such organisms. However, (+) variety was less inhibitory than the other two. Strains of *S. enterica* serovar Typhimurium were inhibited at 500 µg/ml of all the compounds. *L. monocytogenes* NCTC 7973, NCTC 10351, NCTC 11994 were inhibited at 200µg/ml of racemic and (+) forms and at 100µg/ml of (−) form. The strains of klebsiellae, *P. aeruginosa* and *L. monocytogenes* AMRI 3 were highly resistant to all the compounds.

#### UV Spectrophotometer analysis

**Figure 1:**
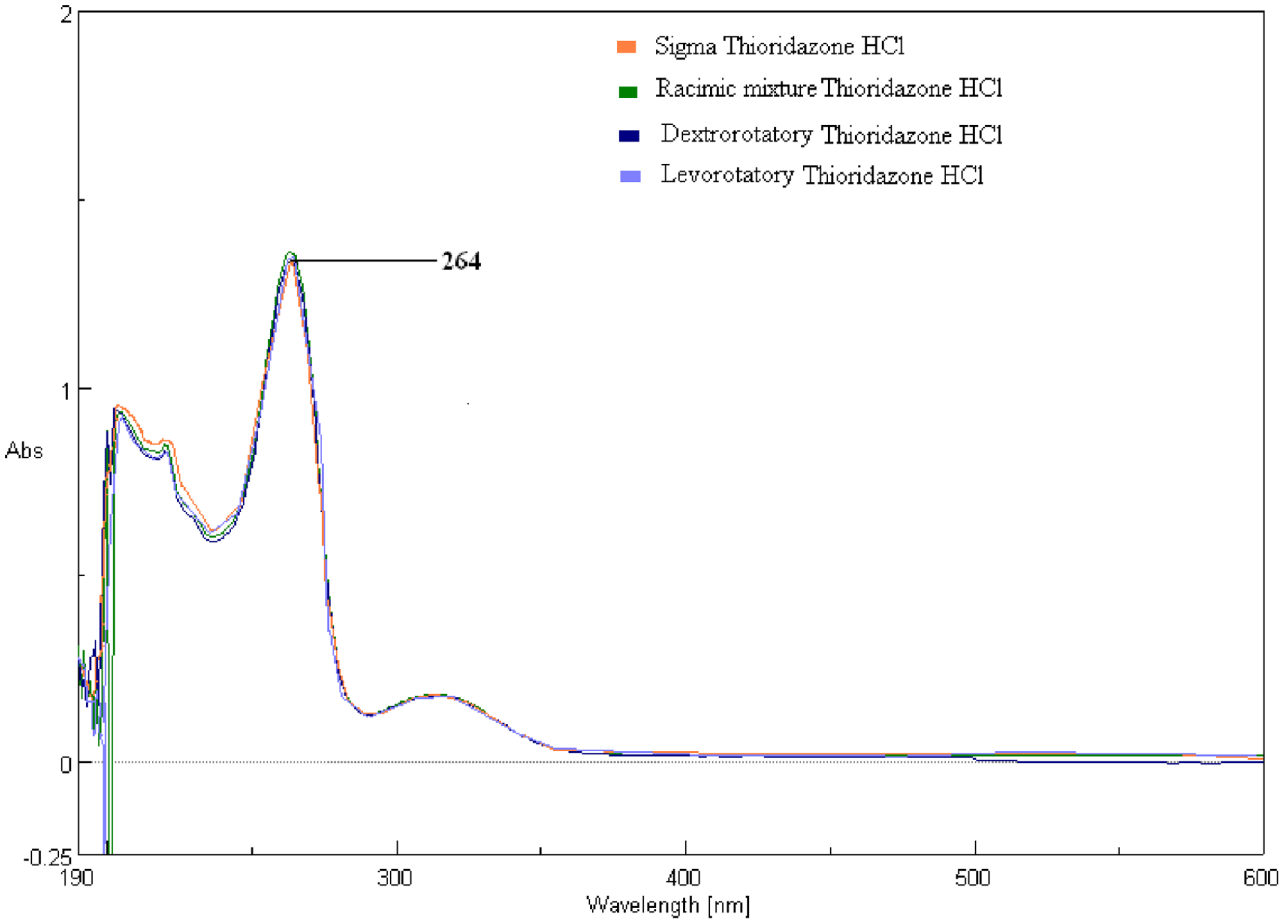
The spectrophotometric scanning results show that all four compounds had the same λ_max_ (264 nm). HPLC result: The chromatogram of the four different samples having the same retention time about 4.6 mints indicating that the compounds are identical except for chirality.

Maximum absorbance (λ_max_) of the 3 forms of Tz and the reference thioridazine (Sigma) were found to be 264 nm. All the four analytes had the same absorbance as well as identical absorption spectra (Fig. 1).

#### HPLC analysis

**Figure 2:**
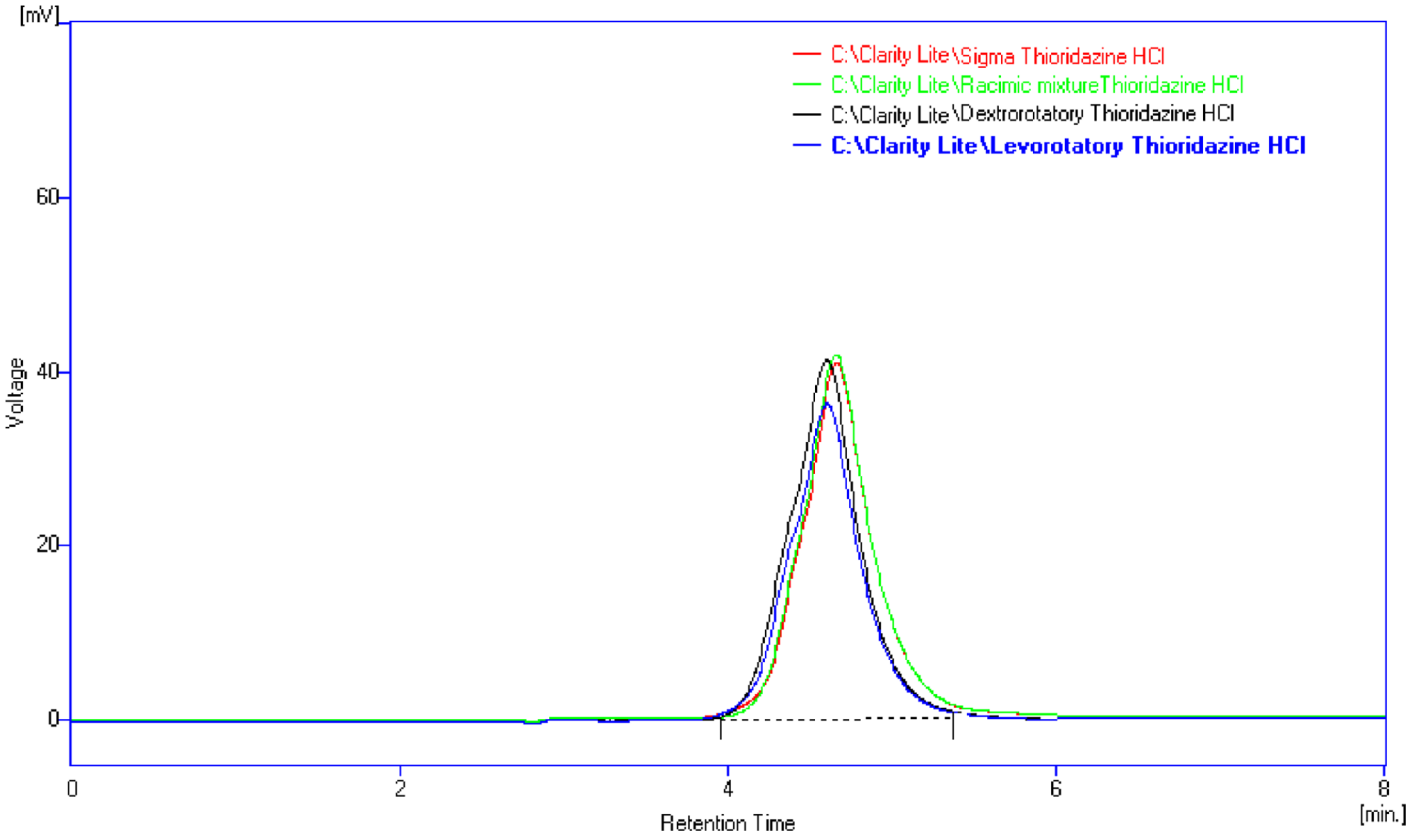
The chromatogram showing that all four different samples had the same retention time (about 4.6 min).

The chromatogram of the three forms of Tz and the reference Tz from Sigma had the same retention time (4.6 min). All the chromatograms were identical showing peaks at the same concentration (Fig. 2).

### *In vivo* Experiments

Virulence of the infection produced by *S. enterica* NCTC 74 is being presented in Table 2. In a control group of 60 mice that received only the challenge the mortality was 86.7%. As the number of CFU of *S.enterica* 74 injected intraperitoneally into mice increased, the % mortality increased, becoming 100% with a dose of 0.95×10^9^ CFU (Table 2). The protective capacity offered by the 3 different forms of Tz shows that there was 100% survival with 200µg/mouse with the (−) form. With the (+) variety the survival was 70% with 200 µg/mouse dose. However, racemic proved to be better than (+) as there was 95% survival with 200µg/mouse dose (Table 2). It may be mentioned here that the animals that received (−) form went to sleep within a few minutes after intraperitoneal injections. Animals of the other two forms went to sleep after 30–40 minutes of intraperitoneal injection of the compounds.

**Table 2:** Effects of 3 forms of thioridazine (Tz) [racemic, (+), (−)] on survival of mice challenged with *Salmonella enterica* serovar Typhimurium NCTC 74^a^.

Control group (not receiving Tz)			Test groups (receiving Tz)			
Saline (ml/mouse)	No. of mice died (N = 60)	Mortality %	Tz (µg/mouse)		No.of mice died (N = 20)	% survived
0.1	52	86.7	Racemic	100 200*	9 1	55 95
			(+)	100 200	12 6	40 70
			(−)	100 200*	7 0	65 100

a Mice received a challenge of 0.95×10^9^ colony-forming units of *S. enterica* NCTC 74 in 0.5 ml of brain-heart infusion medium.

* p<0.001 vs. controls (χ^2^ test).

The tests on the 3 forms of Tz in mice infected with *S.enterica* 74 revealed that 5 animals which received only saline and challenge had >10^8^ live cells in liver, spleen and blood after 18 hr infection. However, the number of CFUs in all the organs were between 10^3^ and 10^4^, being much less in the test batches of mice that received one of the 3 drugs along with challenge. The data were statistically significant (Table 3).

**Table 3:** In vitro activity of sera obtained from blood and homogenates of liver and spleen of mice treated for *Salmonella enterica* 74.

Group	Treatment	CFU/ml^b^		
		Sera		Homogenate
		Heart blood	Liver	Spleen
Control	0.1 ml of a sterile saline	1.8×10^8^ to 5.9×10^8^	6.5×10^8^ to 9.2×10^8^	1.6×10^8^ to 8.7×10^8^
I	200 µg of Racemic form	9.0×10^4^ to 6.6×10^5^	2.8×10^3^ to 3.8×10^4^	2.5×10^3^ to 4.8×10^4^
II	200 µg of (+) form	3.6×10^4^ to 9.6×10^5^	2.2×10^4^ to 5.0×10^5^	7.8×10^3^ to 2.8×10^5^
III	200 µg of (−) form	1.0×10^3^ to 8.0×10^5^	2.0×10^3^ to 4.7×10^4^	1.8×10^3^ to 5.7×10^4^

a Mice received a challenge of 0.95×10^9^ colony-forming units of *S. enterica* NCTC 74 in 0.5 ml of brain-heart infusion medium.

* p<0.001 vs. controls (‘t’ test).

## Discussion

Results obtained in the present study show that racemic, (+) and (−) forms of Tz did not have much difference in their *in vitro* action, only except that (−) form had shown slightly better inhibitory activity than the other two. Standard strains of *L.monocytogenes*, eg. NCTC 7973, 10351, 11994 could be inhibited at 500 µg/ml of the 3 forms of Tz, while *L.monocytogenes* AMRI 3 that was isolated from an acute systemic infection in Kolkata was highly resistant to all the drugs. Both the spectrophotometric and HPLC studies carried out with the 3 forms along with standard thioridazine from Sigma Chemicals, Denmark showed that there was no difference in the λ_max_ and that absorption spectra were identical.

This study further revealed that administration of any form of Tz successfully protected the mice infected with virulent *S.enterica* from lethality. The protection offered by the drugs were also statistically significant as evidenced by the reduction in the viable cell count in the organs of infected mice compared to the animals that were not administered any drug.

Intraperitoneal infection by *S.enterica* in mice is likely to cause phagocytosis by neutrophils [24]. According to Gunn [26] salmonellae can efficiently resist the action of hydrolases due to the action of PmrA/B regulon responsible for inactivation of hydrolases. The MIC of all the compounds with respect to *S.enterica* 74 was 500 µg/ml and is equivalent to weight of water, The amount of Tz forms in a mouse receiving 200 µg dose each would be equivalent to 10 µg/ml, which is one-twentieth of the actual MIC value. Such a distinct protection by Tz forms in mice may be explained by the studies of Ordway *et al* [27].Since these authors could demonstrate that phenothiazines get concentrated 100 fold inside macrophages maintained in a suitable medium, it may be possible that the concentration takes place inside the lysozome leading to rupture of bacterial cell wall. Furthermore as the phenothiazines are known to promote loss of 55 kD protein [28], there may have been a significant reduction of virulence of bacterial cells in the phagolysozome and hence the lethality might have diminished distinctly. In absence of a direct proof regarding the actual mechanism of action of the different forms of Tz, the protection offered by these compounds remains an assumption. Although it may not be possible to recommend Tz alone against bacterial infections on the basis of our observation in the present study, it may be suggested that structural modifications of the original Tz molecule may open up an avenue on the possibilities of producing highly potent protective antibacterial agents in course of time.
